# Tax authorities' interaction with taxpayers: A conception of compliance in social dilemmas by power and trust

**DOI:** 10.1016/j.newideapsych.2014.12.001

**Published:** 2015-02

**Authors:** Katharina Gangl, Eva Hofmann, Erich Kirchler

**Affiliations:** University of Vienna, Faculty of Psychology, Austria

**Keywords:** Coercive power, Legitimate power, Reason-based trust, Implicit trust, Tax compliance, Cooperation

## Abstract

Tax compliance represents a social dilemma in which the short-term self-interest to minimize tax payments is at odds with the collective long-term interest to provide sufficient tax funds for public goods. According to the Slippery Slope Framework, the social dilemma can be solved and tax compliance can be guaranteed by power of tax authorities and trust in tax authorities. The framework, however, remains silent on the dynamics between power and trust. The aim of the present theoretical paper is to conceptualize the dynamics between power and trust by differentiating coercive and legitimate power and reason-based and implicit trust. Insights into this dynamic are derived from an integration of a wide range of literature such as on organizational behavior and social influence. Conclusions on the effect of the dynamics between power and trust on the interaction climate between authorities and individuals and subsequent individual motivation of cooperation in social dilemmas such as tax contributions are drawn. Practically, the assumptions on the dynamics can be utilized by authorities to increase cooperation and to change the interaction climate from an antagonistic climate to a service and confidence climate.

## Highlights

• Cooperation is determined by the dynamic between power and trust.
• We distinguish between coercive and legitimate power of authorities.
• We distinguish between reason-based and implicit trust in authorities.
• We describe how interaction climates and cooperation can be changed.

## 1 Introduction

Citizens appreciate public goods such as schools or hospitals. Funding the public goods through taxpaying, however, represents a social dilemma in which the individual short-term interest to minimize paying taxes is at odds with the long-term collective interest to ensure sufficient tax payments for financing the public goods (Balliet & Van Lange, 2013). To overcome the social dilemma and to insure high tax compliance among citizens, tax authorities rely on two measures. Power measures such as audits and fines and trust related measures such as fair procedures (e.g., Allingham & Sandmo, 1972; Feld & Frey, 2007; Srinivasan, 1973). In research, the positive impact of both measures on tax compliance received empirical support (e.g., Muehlbacher & Kirchler, 2010; Wahl, Kastlunger, & Kirchler, 2010).

Surface validity might suggest that power and trust are incompatible and the opposites of each other. In contrast, we assume that power and trust are related in a specific dynamic in which they mutually destroy or mutually foster each other and in turn influence tax compliance. However, distinct theoretical assumptions about the dynamics between power and trust are missing. The purpose of the present theoretical paper is to conceptualize these dynamics and to elaborate on how they might influence tax compliance. This conceptualization serves as the theoretical basis for empirical research and conclusions how to increase tax compliance in particular and cooperation in social dilemmas in general.

There is little doubt that audits and fines are necessary to levy taxes, however, they are not the only determinants to ensure contributions. Experiments on tax behavior in the laboratory have consistently supported the positive impact of audits and fines on compliance (Blackwell, 2007). Nonetheless, the effects are rather weak. Field studies and surveys have yielded effects that are lower than, and sometimes the opposite of the predicted effects (e. g., Andreoni, Erard, & Feinstein, 1998). Additionally, Feld and Frey (2007) question whether audits and fines may destroy trust, as they crowd out the intrinsic motivation to cooperate among committed and cooperative citizens. Thus, besides “economic” determinants such as audits and fines, “psychological” determinants such as the motivation to comply, the attitudes of taxpayers towards the state, the government and taxation, transparency and understanding of tax laws, personal and social norms, and fairness perceptions were shown to impact tax compliance (Braithwaite, 2003; Kirchler, 2007; Torgler, 2003).

Kirchler (2007) and Kirchler, Hoelzl, and Wahl (2008) endeavored to integrate the economic and psychological factors into a comprehensive two-dimensional framework, the Slippery Slope Framework (SSF). The dimension power of authorities aggregates economic determinants and is defined by taxpayers' perception of authorities' capacity to detect and punish tax evaders. The dimension trust in authorities covers psychological bases of tax compliance and results from taxpayers' general opinion that the tax law and regulations are clear and easy to follow, and that the tax authorities operate fairly and benevolently in the interest of the community. The SSF asserts that both the power of authorities and the trust in authorities can solve the social dilemma of tax compliance.

**Fig. 1:**
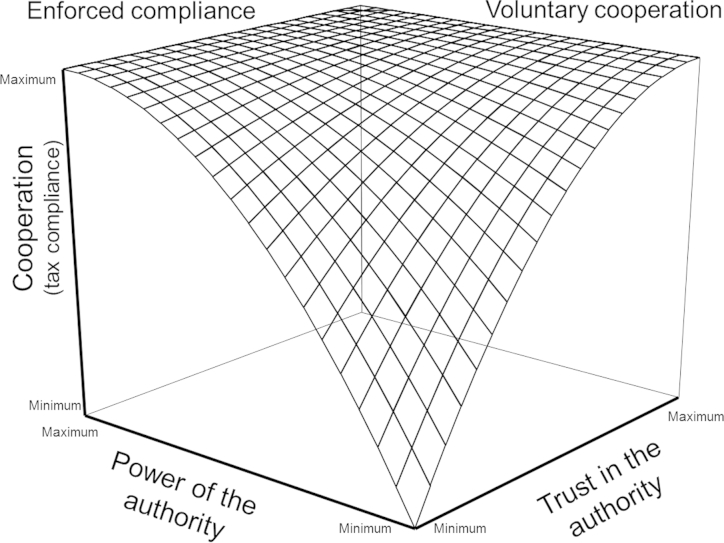
The Slippery Slope Framework (Kirchler, Hoelzl, and Wahl, 2008, p. 212).

On the individual taxpayer level, the framework differentiates between two motivations to comply with tax law, enforced compliance and voluntary cooperation. Enforced compliance results from the power of tax authorities, whereas voluntary cooperation is driven by the taxpayers' trust in tax authorities. On the aggregate level, the SSF postulates that power and trust define different interaction climates between tax authorities and taxpayers: while the exertion of strong power by the authorities fosters an antagonistic climate, high trust is the prerequisite of a synergistic climate (Kirchler, 2007; Kirchler et al. 2008). Fig. 1 depicts power and trust as independent dimensions, positively related to enforced compliance and voluntary cooperation, respectively, and to an antagonistic and synergistic climate, respectively.

Empirical evidence generally supports the relevance of power and trust as determinants of compliance (Kogler et al., 2013; Muehlbacher & Kirchler, 2010; Muehlbacher, Kirchler, & Schwarzenberger, 2011; Wahl, Endres, Kirchler, & Böck, 2011; Wahl et al. 2010). For instance, in a representative sample of self-employed taxpayers, trust and power co-varied with tax compliance (Muehlbacher & Kirchler, 2010). Kogler et al. (2013) and Wahl et al. (2010) found that compliance is highest if both power *and* trust are perceived as high. This result suggests an additive effect of power and trust. Moreover, a dynamic relationship between power and trust can be assumed.

In the conceptualization of the SSF, Kirchler et al. (2008) speculate about a dynamic relationship but they offer no elaboration of the possible interaction effects between power and trust. In contrast to surface validity, which might suggest that power and trust are incompatible, they assume that power and trust might not only weaken but also strengthen each other. So far, empirical studies in the tax behavior context suggest that power and trust are influencing each other positively (Kogler et al. 2013; Muehlbacher et al. 2011; Wahl et al. 2010). Nevertheless, in various research fields the theoretical conceptualization and the empirical evidence for the mutual effects of power and trust are inconsistent, which suggests that there is both a fostering as well as an eroding influence of power on trust (Adler, 2001; Bijlsma-Frankema & Costa, 2005; Das & Teng, 1998; Kumlin & Rothstein, 2005; Möllering, 2005). This inconsistency may originate from different conceptualizations of power and trust and from diverse operationalizations in empirical investigations. Therefore, we propose to distinguish between the independent qualities of coercive power and legitimate power. We further differentiate between reason-based trust and implicit trust. These distinctions will provide an explanation of the dynamics between power and trust.

The aim of the present paper is to shed light on the effects of the mutual interaction of coercive and legitimate power on the one hand, and reason-based and implicit trust on the other hand, as well as to formulate assumptions regarding the consequences on the interaction climate between tax authorities and taxpayers as well as on tax compliance. Consequently, we extend the SSF by distinguishing between three types of interaction climates resembling for instance Alm and Torgler's (2011) interaction styles and respective qualities of cooperation comparable to Kelman's (2006) psychological processes of social influence. Hence, we not only integrate the dynamics between power and trust in well-established existing theories but more importantly show how these dynamics can be used to transform a hostile interaction into an interaction in which voluntary and committed cooperation prevails.

We extend the SSF borrowing from the literature on social dilemmas, social influence, organizational behavior, and leadership; hence, we make predictions beyond tax compliance on general interaction climates, motivations to cooperate, and eventually, contributions to public goods which are regulated by authorities such as insurance funds, public transportations, or business organizations. All these cases represent social dilemmas, similar to the social dilemma of tax compliance, in which the short-term self-interest is at odds with longer-term collective interest (Van Lange, Joireman, Parks, & Van Dijk, 2013). Each single individual would be better off by not contributing to the public good but nonetheless taking advantage of the public good provision (Dawes, 1980; Ostrom, 2000). However, if all individuals would chose this strategy no public good would be provided and eventually, all would end up worse off than if all had cooperated (Dawes, 1980). Authorities, however, as intermediates are one possibility overcoming this tragedy of the commons by actively regulating and monitoring the individual contributions to the public good (Van Vugt, 2009; Van Vugt & De Cremer, 1999). Hence, although our predictions on the dynamics between power and trust are focused on tax authorities interacting with taxpayers, we propose, that these predictions apply to all authorities regulating individuals' contributions to public goods.

In the remainder of this paper we first introduce the concepts of coercive power and legitimate power, and reason-based trust and implicit trust. Second, we speculate on the dynamics between the different qualities of power and trust and how these impact tax compliance. Third, we discuss the consequences of different qualities of power and trust for interaction climates and the respective motivations to comply. Fourth, the paper concludes with observations on the transformation from one type of interaction climate to another.

## 2 Qualities of power

Power has received much attention in various scientific disciplines. Besides specific perspectives taken by different disciplines, there is considerable agreement on a general definition of power. Power is consistently defined as the potential and perceived ability of a party to influence another party's behavior (e.g., Freiberg, 2010; French & Raven, 1959; Molm, 1994).

In research on the regulation mechanisms of citizens' behavior, two competing theories of power are widely recognized, the conceptualizations of coercive and legitimate power. The perspective on coercive power is based on Becker's (1968) economic approach which argues for strict control and punishment to influence individuals' utility functions and in turn, their behavior. The second and more recently developed approach by Tyler (2006) argues that legitimate power, i.e., the power of accepted authorities, is more appropriate and effective in shaping individuals' behavior than severe controls and punishment.

We seek to integrate both perspectives of power in the SSF and refer to the social-psychological theory of the ”bases of social power” developed by French and Raven (1959), and Raven (1965). The bases of social power were initially conceptualized to explain relations between supervisor and employee, i.e. individuals. It can, however, be assumed that people's behavior in organizations, public institutions, and the state is shaped by the same perceptions and judgments of the dominant party as in bilateral relationships or small group settings (Tyler, 2006). French and Raven's approach distinguishes between coercive power, reward power, legitimate power, expert power, referent power, and information power. The different bases of power are seen as independent implying that authorities cannot only hold one of the bases of power but several bases of power at the same time. Moreover, the different bases of power can be integrated into a two-dimensional structure (Raven, Schwarzwald, & Koslowsky, 1998): the six bases of power fall into the two independent categories of harsh and soft forms of power. To be consistent with the terminology in the context of the regulation of citizens' behavior (Turner, 2005), we use the term coercive power for harsh power and legitimate power for soft power. In the following, the terms coercive power and legitimate power refer to our conceptualization and not to French and Raven's (1959; Raven, 1965) terminology.

Perceived coercive power originates from the pressure applied through either punishment or remuneration. Our concept incorporates the two harsh forms of social power bases, i.e., coercive power and reward power. Whereas coercive power is based on the expectations of the influenced party that non-cooperative behavior will be punished (e.g., through monetary penalties or imprisonment), reward power operates through the expectations of the influenced party that obeying the rules of the powerful party will be rewarded (e.g., through awards or gratuities). Our concept of coercive power is consequently based on incentivizing and compulsion. Individuals who do not obey the rules of the authorities will face monetary, physical, social, or psychological costs (e.g., being fined or not receiving a reward, being excluded from future transactions).

Perceived legitimate power originates from legitimization, knowledge, skills, access to information, and identification with the powerful party, and comprises French and Raven's (1959) soft forms of power, namely legitimate power, expert power, information power, and referent power. Legitimate power operates through the accepted right to influence others by means of, for instance, agreed election rules, the norm of reciprocity (Gouldner, 1960), social responsibility, and equity norms (Berkowitz & Daniels, 1963). Expert power operates through the attribution of knowledge and skills that leads to the perception that the expert has a high capacity to lead (Raven, 1992, 1993). Information power is based on sharing of valued information (Raven, 1965, 1992, 1993). Referent power results from the dependent party's identification with the influencing party (Raven, 1992, 1993). Our concept of legitimate power is based on the fact that the legitimate authorities use information, charisma, legitimization, and expertise to convince taxpayers that it is the right course of action to cooperate.

Hence, in contrast to the conceptualization of Becker (1968) and Tyler (2006), in the current conceptualization the qualities of power are multifaceted. Coercive power includes not just deterrence but also audits, punishment and rewards, and legitimate power comprises not just acceptance of the authorities but also the legal position, distribution of information, identification with the authorities, and their expertise. Importantly, in our conceptualization of power, coercive power and legitimate power are not seen as opposing entities but as independent factors (Hofmann, Gangl, Kirchler, & Stark, 2014). Authorities can wield coercive power without legitimate power, legitimate power without coercive power as well as they can wield both qualities of power at the same time (Hofmann et al. 2014). Accordingly, the tax authorities can or cannot be perceived as having the means to punish and reward taxpayers and can or cannot be perceived as having procedural measures to make it acceptable and easy for taxpayers to contribute.

## 3 Qualities of trust

The importance of trust in social systems is broadly recognized. Despite notable differences in approaching the phenomenon of trust, there is wide agreement on defining trust as the willingness of a party to take a risk (Lewis & Weigert, 1985a) and “to be vulnerable to the actions of another party based on the expectation that the other will perform a particular action important to the trustor, irrespective of the ability to monitor or control that other party” (Mayer, Davis, & Schnorrman, 1995, p. 712).

Two independent qualities of trust are distinguished: trust based on cognitive-rational processes and trust based on automatic-affective processes (Castelfranchi & Falcone, 2010; Lewis & Weigert, 1985a; McAllister, 1995; Nooteboom, 2002; Tyler, 2003). We draw on Castelfranchi and Falcone's (2010) conceptualization of trust and differentiate between reason-based and implicit trust. Reason-based trust corresponds to concepts of calculative trust (Coleman, 1994; Fehr, 2009), rational trust (Ripperger, 1998), and knowledge-based trust (Lewicki & Bunker, 1996). Implicit trust corresponds to concepts of identification-based trust (Tyler, 2001), habitus trust (Misztal, 1996), social trust (Welch et al. 2005), and affective trust (Jones, 1996).

Reason-based trust results from a deliberate (rational) decision grounded on four criteria: goal achievement, dependency, internal factors, and external factors (Castelfranchi & Falcone, 2010). First, the trustor evaluates whether the other party is pursuing a goal that is important to the trustor. Second, it is evaluated whether the trustor depends on the other party. Third, a positive evaluation of internal factors of the other party, i.e., competence, willingness, and harmlessness, is required. Fourth, the external factors in decision-making include the perception of opportunities and dangers. In this sense, reason-based trust corresponds to trust developed by a rational agent who trusts that there are good reasons to expect the other will forgo opportunistic goals (Coleman, 1994; Fehr, 2009; Mayer et al. 1995).

Implicit trust is defined as an automatic, unintentional, and unconscious reaction to stimuli (Castelfranchi & Falcone, 2010). The automatic reaction originates from associative and conditioned learning processes and memory and is expected to emerge in situations in which shared social identities are activated (Castelfranchi & Falcone, 2010; Coulter & Coulter, 2002). Social categories or groups serve as stimuli which provoke the perception that certain social practices and norms can be relied on and that every person, organization, or authority that falls into this category can be trusted (Castelfranchi & Falcone, 2010; Lewis & Weigert, 1985b; Messick & Kramer, 2001). It can be expected that an authority perceived as belonging to the same category like the taxpayer will be evaluated positively and implicit trust should be higher when compared to trust in authorities perceived as belonging to another category (Tanis & Postmes, 2005). In addition to social identities also the cue that the tax authorities are an official institution might serve for some as a sign which activates automatic trust. Having repeatedly successfully interacted with public institutions leads to automaticity in the interaction (Verplanken, 2006) and a situation in which implicit and habitual trust in the institution prevails (Misztal, 1996). Other cues which activate implicit trust might be signs of warmth in contrast to hostility or cooperation in contrast of competition communicated through tax authorities' communication (websites, brochures, buildings; Williams & Bargh, 2008). To conclude, implicit trust occurs without the conscious recognition of reasons to trust and thus, without considering competence or intention of the official institution.

The different conceptions of trust correspond to the two-process theories of cognition in which it is distinguished between system 1 and system 2 (Kahneman, 2003; Sherman, Gawronski, & Torpe, 2014). System 1 is working fast, effortless, associative and often is emotionally charged, governed by habit and difficult to control and modify. System 2 is based on slow, effortful, serial and deliberately controlled cognition, relatively flexible and potentially rule-governed (Evans, 2008; Kahneman, 2003). Whereas system 1 describes the functionality of implicit trust, system 2 explains the mechanism of reason-based trust. However, the two-process theories also assume that reason-based trust and implicit trust are related (Evans, 2008). Depending on the circumstances, the two qualities of trust might have parallel as well as sequential relationships (Evans, 2008). For instance, reason-based trust and implicit trust are operating together if taxpayers might implicitly trust the tax authorities because of cues such as a friendly voice on the tax line and at the same time might gain reasons to trust as the same person on the tax line also offers a competent advice. On the other hand, reason-based trust and implicit trust might operate independently, if taxpayers are cognitively too lazy to consider whether the tax authorities give reasons to trust, and rather just implicitly trust without questioning the tax authorities as official institution. Taxpayers also might in principle mistrust official institutions and hence, only trust the tax authorities, if they have proven evidence that the tax authorities act benevolently and competently. For a sequential relationship, research suggests that after taxpayers gained relevant experience based on deliberatively considering tax authorities' trustworthiness, reason-based trust enhance or even changes its quality to fast, and implicit trust (Evans, 2008; Sun, Slusarz, & Terry, 2005). Thus, in the long-run implicit trust develops with increasing reason-based trust that in the end becomes implicit trust.

## 4 Dynamics between qualities of power and trust

**Fig. 2:**
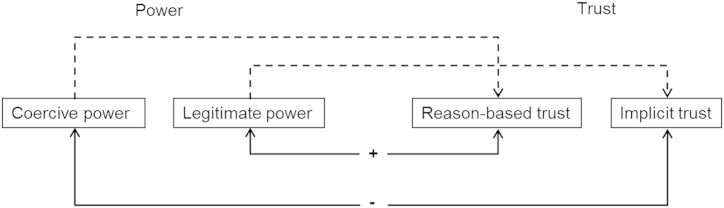
Dynamics between qualities of power and qualities of trust.

Depending on the quality of power and the way power is exerted and perceived, trust in the powerful party can either be strengthened or weakened (e.g., Castelfranchi & Falcone, 2010; Choudhury, 2008; Korczynski, 2000; Kumlin & Rothstein, 2005). Also the quality of trust can affect the perception of authorities' power. In the SSF, Kirchler et al. (2008) conclude that tax authorities which enforce compliance through hostile and coercive measures run the risk of losing trust, whereas tax authorities perceived as legitimate may gain trust and the voluntarily cooperation of trustors. Also, the SSF proposes that if the authorities gain trust, they also enhance their legitimate power (Kirchler et al. 2008). In this vein, we assume two strong mechanisms which in general regulate the dynamics between power and trust: coercive power and implicit trust mutually decrease each other and that legitimate power and reason-based trust mutually increase each other. Additionally, we propose two second order relationships such as that coercive power and reason-based trust are related to each other via legitimate power and that legitimate power and implicit trust are related through reason-based trust. In the following, these assumptions are presented in detail (Fig. 2).

*Coercive power and implicit trust are mutually decreasing each other.* If coercive power manifests by strict controls and fines, particularly if addressed at the individual, it provokes deliberate reasoning regarding possible gains and losses and the risk of non-compliance and therefore interjects and destroys implicit trust (Kirchler, 2007). Additionally, coercion may weaken affective and social bonds and interrupt habitual cooperation (Balliet, Mulder, & Van Lange, 2011; Castelfranchi & Falcone, 2010; Kramer, 1999; Nooteboom, 2002; Tenbrunsel & Messick, 1999). Coercive power damages implicit trust and social bonds because asymmetrically established control mechanisms indirectly convey the message that the person to which coercive power is addressed, is not trusted (Das & Teng, 1998; Nooteboom, 2002). As a reaction, implicit and automatic trust cannot emerge and instead coercive power is assumed to lead to reactance and deliberate and strategic reasoning (Balliet et al. 2011; Kirchler, 1999; Kirchler et al. 2008).

However, implicit trust also reduces coercive power. People who trust implicitly base their automatic trust on shared norms, signaled values, and habits. Accordingly, audits and fines, which are expressions of coercive power, are not perceived as necessary (Cummings & Bromiley, 1996; Dekker, 2004; Yamagishi, 1988). Implicit trust activates social control mechanisms and relational governance (Dekker, 2004), and it fosters spontaneous, unreflected cooperation (Castelfranchi & Falcone, 2010). Willingness to spontaneously cooperate with another party reduces the complexity of the social world (Luhmann, 2000), because control is not necessary (Das & Teng, 1998; Inkpen & Currall, 2004). Hence, those who implicitly trust might not demand tax authorities to increase their coercion.

*Legitimate power and reason-based trust are mutually amplifying each other*. Legitimate power and reason-based trust are strongly entwined and can be seen as the two sides of the same coin. Hence, their mutual fostering influence is not only theoretically but also empirically well established (Bijlsma-Frankema & Van de Bunt, 2002; Das & Teng, 1998; Malhotra & Murninghan, 2002; Mulder, Van Dijk, De Cremer, & Wilke, 2006). There are several reasons for taxpayers to trust; parties with legitimate power are perceived as competent to provide assistance and support (Bijlsma-Frankema & Van de Bunt, 2002; Castelfranchi & Falcone, 2010). Additionally, legitimate processes can provide a “track record” of the behavior of the parties involved and thereby build up a positive reputation (Das & Teng, 1998). Hence, legitimate power provides reasons to trust the tax authorities.

At the same time, reason-based trust also increases legitimate power because reason-based trust both emerges from and leads to the recognition of the legitimacy of the authorities and the acceptance of the authorities (Castelfranchi & Falcone, 2010). If the perception of shared goals prevails, it is likely that the party is also accepted as the rightful authority (Inkpen & Currall, 2004). Reason-based trust permits and requires the powerful authorities to influence taxpayers' behavior (Cullen, Johnson, & Sakano, 1995; Das & Teng, 1998).

*Coercive power and reason-based trust are related through legitimate power*. There are reasons why coercive power is destroying trust, for instance, if it is applied without competence and there are reasons why coercive power can strengthen trust, for instance, if coercive power is perceived as being used competently only against tax evaders. Hence, coercive power in combination with legitimate power has a relationship with reason-based trust. It is assumed that authorities which are perceived wielding coercive and legitimate power strengthen reason-based trust, whereas authorities perceived as wielding coercive power and not legitimate power reduce reason-based trust. Coercive power which is perceived to be used in a legitimate way, for instance, in a procedural fair manner to effectively inhibit rule-breaking behavior (Bachmann, Knights, & Sydow, 2001; Hofmann et al. 2014; Van Prooijen, Gallucci, & Toeset, 2008), gives good reasons to trust. Hence, coercive power combined with legitimate power is assumed to be perceived as targeted to evaders and as a safeguard of honest taxpayers which fosters reason-based trust. In contrast, high coercive power combined with low legitimate power destroys reason-based trust because the authorities are perceived to wield audits and fines in an incompetent and random way and even might be seen to prosecute honest taxpayers. If coercive power is low and legitimate power is high, reason-based trust also is high, as the wielding authorities are perceived to lead through legitimacy alone. If both, coercive power and legitimate power are perceived to be low also reason-based trust is low. The authorities are not legitimated and weak; hence, they are not seen as capable to guarantee a fair tax system. As the impact of coercive power on reason-based trust depends on legitimate power, coercive power which is wielded without highlighting its legitimacy likely reduces reason-based trust in the tax authorities. In general, coercive power and reason-based trust are related over the perception of legitimate power.

*Legitimate power and implicit trust are related through reason-based trust*. Legitimate authorities increase first, reason-based trust and second, through establishing a stable system of functioning cooperation also implicit trust. Trust initially based on rational consideration transforms into implicit trust through routine. The more routine taxpayers can develop interacting with authorities perceived as competent the more reason-based trust gradually changes into implicit trust.

The different qualities of power and trust are assumed to be perceived by taxpayers in a specific way. Legitimate power and reason-based trust are assumed to be positively related, whereas coercive power and implicit trust are negatively related. These dynamics are assumed to hold not only for vertical relationships in which tax authorities are seen to affect a specific taxpayer's behavior but also for horizontal relationships in which a taxpayer's behavior is affected by the observation on how the tax authorities treat other taxpayers not the taxpayer in question.

The relationship of qualities of power and trust holds not only, if power is perceived to be wielded on a taxpayer, but also if it is wielded on other taxpayers. Coercive power perceived to be directed at the individual reduces implicit trust, however, also if it is perceived to be directed to other taxpayers it may fuel the impression that a considerable number of taxpayers tries to evade taxes, hence, that the social norm of tax honesty is low. Accordingly, coercive power indirectly conveys the message that the other taxpayers cannot be trusted to pay their fair share of taxes and therefore need to be enforced (Mulder et al. 2006). Also legitimate power addressed to other taxpayers has the same effect compared to when it is addressed on the individual. Addressing the individual with legitimate power already conveys a message about the other taxpayers. Legitimate power is applied competently which means it is addressed in general to all taxpayers which fosters reason-based trust. Coercive power and legitimate power addressed to other taxpayers will increase reason-based trust because it leads to the perception that the right people, those who try to evade, are controlled whereas the honest taxpayers are protected.

To sum up, two main mechanisms are assumed to determine the relationship between power and trust, a negative relationship between coercive power and implicit trust and a positive relationship between legitimate power and reason-based trust. Additionally, it is assumed that coercive power is independent from reason-based trust and that legitimate power is positively related to implicit trust. Whereas coercive power impacts reason-based trust only due to its perceived legitimacy, legitimacy is assumed to steadily increase implicit trust by increasing reason-based trust.

Based on these assumptions and the empirical evidence provided by Kogler et al. (2013) and Wahl et al. (2010) we propose that the manipulation of high power and high trust was in fact a manipulation of coercive power and legitimate power. Whereas the manipulation of high power and low trust was likely perceived as coercive power applied with low competence to guarantee a fair system, low power and high trust were likely perceived as legitimate power without the means to enforce compliance with the law. Hence, only high power and high trust, thus, high coercive power and high legitimate power were perceived as a competent safeguard of cooperation which induced the overall highest compliance rates.

## 5 The impact of the dynamic of power and trust on tax climate and tax cooperation

Based on the presented assumptions regarding the dynamics between power and trust we extend the SSF and distinguish on the aggregated level between three interaction climates (cf. Alm & Torgler, 2011): an antagonistic climate, a service climate and a confidence climate. This differentiation is based on conceptualizations from organizational research distinguishing between market or price mechanisms regulating social interactions, authorities, hierarchy-based or bureaucratic mechanisms of regulation and finally, community or trust mechanisms of managing social interactions (Adler, 2001; Bradach & Eccles, 1989; Haslam & Fiske, 1999; Ouchi, 1979). We also hypothesize that the different interaction climates lead, in the long run, on the individual level to corresponding forms of cooperation by taxpayers: enforced tax compliance, voluntary tax cooperation, and committed tax cooperation. Again, current assumptions are in line with earlier research on social influence for instance by Kelman (Kelman, 1961, 2006), who concluded that three psychological processes determine individual reactions to influence: compliance, identification, and internalization. Compliance with rules is based on incentives, identification with roles is grounded in reciprocity and modeling and internalization of values is based on value congruence or perceived continuity of the own self-concept (Kelman, 2006; Kelman & Hamilton, 1989). Hence, in the extended SSF, we conclude that the dynamics between power and trust are the preconditions of three cooperative climates, the antagonistic, the service, and the confidence climate, with corresponding qualities of motivations to cooperate, enforced compliance, voluntary cooperation and committed cooperation.

**Fig. 3:**
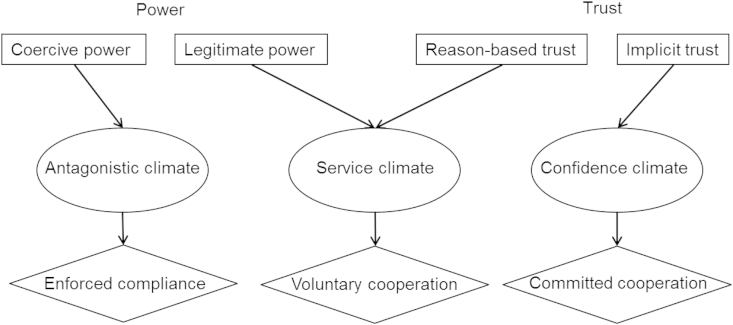
Dynamics between power and trust affecting climates of cooperation and motivations to comply.

The conceptualization of the interaction between authorities and individuals builds on the classical psychological insight, that authorities' actions create a specific social atmosphere, hence a cooperative climate which in turn provokes on the individual level specific corresponding habitual reactions of cooperation (Lewin, Lippitt, & White, 1939; Schneider, 2013). Fig. 3 summarizes our assumptions and shows that coercive power favors an antagonistic climate and enforced compliance, whereas legitimate power and reason-based trust are the antecedents of a service climate and voluntary cooperation. Implicit trust is the base of a confidence climate and committed cooperation.

In the antagonistic climate coercive power prevails and a “cops and robbers” attitude is predominant with taxpayers and tax authorities working against each other (Kirchler et al. 2008). Tax authorities perceive taxpayers as “robbers” who try to evade and escape the tax authorities. In turn, taxpayers may feel prosecuted and harassed by the tax authorities (“cops”) and may feel the necessity to “hide”. The antagonistic climate is characterized by mistrust and resentment and leads to a vicious circle in which coercive power and mistrust mutually reinforce each other. Thus, compliance in such a climate needs to be enforced. Enforced compliance is characterized, for instance, by the feeling that tax authorities are interested in catching taxpayers evading, independent of whether the wrongdoing is intended or not (Kirchler et al. 2008). These assumptions received empirical support through experiments showing that high in contrast to low coercive power leads to a perceived antagonistic climate and enforced compliance of taxpayers (Hofmann et al. 2014). The thoughts underlying an antagonistic climate that taxpayers can only be forced to comply with the tax law by dint of controls and fines match the standard economic paradigm of tax behavior (Allingham & Sandmo, 1972). The disadvantage of an antagonistic climate is — besides costly audits — that taxpayers are likely to develop motives of opposition and reactance (Braithwaite, 2009; Kirchler, 2007) which cause instability in tax behavior and tax collection: when the tax authorities lose power, taxpayers lacking the intrinsic motivation to comply are expected to engage in evasion.

The service climate bases on legitimate power and reason-based trust. It is characterized by a “service and client” attitude which means that taxpayers and tax authorities collaborate on the basis of well-defined rules and standards. Tax authorities perceive taxpayers as clients who expect and deserve professional, fair, and supportive services. Taxpayers reciprocate this attitude by contributing their tax share. Taxpayers who perceive the authorities as being supportive and competent are likely to cooperate voluntarily. Voluntary tax cooperation reflects the view of taxpayers that paying taxes is an accepted obligation as well as a necessity if the state is meant to provide public goods (Kirchler & Wahl, 2010; Wahl et al. 2010). These assumptions also received empirical support through experiments conducted with taxpayers showing that high in contrast to low legitimate power leads to a perceived service climate and voluntary cooperation (Hofmann et al. 2014). The advantage of the service climate lies in its stability — a single event of inappropriate services provided by the tax authorities will not lead to reduced taxpayers' cooperation, because the taxpayers themselves want the tax system to work smoothly. A disadvantage of a service climate may be the bureaucracy entailed in producing elaborate written rules as well as complex procedures to treat taxpayers fairly, which results in substantial administrative overheads (Ouchi, 1979).

In a confidence climate implicit trust prevails. Taxpayers automatically trust the tax authorities and cooperate without thinking about it. Taxpayers pay their taxes because they perceive the tax authorities to work on the basis of shared norms and values or simply cooperate out of a habit. Tax authorities on the other hand, reinforce implicit trust by showing respect to the honest taxpayers (Feld & Frey, 2002). Tax authorities perceive themselves as working in the name of the taxpayers; they show empathy and feel obliged to offer support. Taxpayers perceive the tax authorities as working for the good of the community and reciprocate by contributing their share because they feel intrinsic motivation as members of the same community. For taxpayers, tax compliance is a personal and societally shared norm that is binding. Shared perceptions and values prevail and taxpayers are personally committed to the tax system. Committed cooperation is characterized by taxpayers' feelings that paying taxes is a customary thing to do and a moral obligation also followed by fellow citizens. Taxpayers feel committed to the tax system as a whole and actively engage to make the system work. The main advantage of a confidence climate is that taxpayers do not follow the letter of the law, but comply with the spirit of the law. Specific and complicated tax legislation is not needed because taxpayers follow moral standards instead of specific tax rules. According to Sloterdijk (2010), the main benefit of a confidence climate which induces that taxpayers feel committed to contribute their share, is that taxpayers are in a position of self-determination and generosity where they actively participate in a vital democracy and take responsibility for their society. Undoubtedly, a disadvantage of a confidence climate is its vulnerability to free-riders if tax authorities are perceived to avoid controls and punishment of tax evaders (Ouchi, 1979). Adler (2001) adds for the organizational context that such a confidence climate should be reflective and grounded in open dialogue among the interacting parties to avoid blind and traditionalistic loyalty.

## 6 Conclusions

As a main contribution of the present theoretical elaboration on the dynamic between power and trust, it can be derived how authorities such as the tax authorities can change and enhance cooperative climates and cooperation in social dilemmas. A negative dynamic between coercive power and implicit trust and a positive dynamic between legitimate power and reason-based trust explain how tax authorities can solve the social dilemma of taxpaying by either creating an antagonistic climate with enforced compliance, a service climate with voluntary cooperation, or a confidence climate with committed cooperation. In the following, the present paper concludes how a change from one climate to another climate can be accomplished, how the present assumptions can fuel empirical research, and why the dynamic between power and trust explains tax compliance and cooperation in social dilemmas in general.

As a practical implication of the dynamic between power and trust, it is possible to demonstrate how a change from one climate to another emerges and why some interaction styles are more “slippery” than others. Countries differ in their interaction styles with taxpayers and their tax honesty (Alm & Torgler, 2006; Kogler et al. 2013). In some developing countries authorities lack power and trust, hence, a state of instability and in extreme cases a state of anarchy prevails with low levels of compliance, whereas on the other extreme, stable countries such as northern European ones exist displaying high levels of power and trust in the authorities that guarantee high tax compliance (Kogler et al. 2013). Also, on the individual level differences prevail between taxpayers (Braithwaite, 2003). Individuals within a country differ in their motivation to be honest and can be distinguished into taxpayers who perceive low power and hold low trust and hence, intentionally evade, perceive low legitimate power and fail to comply due to, for instance, complex tax laws and complex procedures or perceive high levels of trust and cooperate out of commitment to the community. We are convinced that tax authorities possess the measures to transform, on the aggregate level, a tax climate of distrust gradually into a climate of confidence. On the individual level, taxpayers' enforced compliance can be transformed into voluntary and committed cooperation. In line with the full range leadership model (Avolio & Bass, 1991; Kirkebride, 2006), in which the laissez-fair leadership develops over various stages into transactional leadership based on incentivizing behavior and further into transformational leadership based on a vision and shared values (Antonakis, Avolio, & Sivasubramaniam, 2003; Kirkebride, 2006), we also propose a possible transformation from one interaction climate into another.

Under circumstances of low power and of low trust, tax compliance will be at a minimum, no matter whether it is on a country or individual level. In such a situation, coercive power can be a starting point for tax compliance. Laboratory experiments indicate that in a social dilemma situation with low levels of trust, authorities using coercive power are efficiently increasing compliance (Van Vugt & De Cremer, 1999). However, the psychological effectiveness of coercive power lies in its potential to scare, deter and enforce taxpayers through efficient and strict audits and severe fines. Accordingly, coercive power precludes the emergence of implicit trust. Instead a vicious circle of mistrust and consequently stronger coercive power is likely to develop between the tax authorities and the taxpayers resulting in an antagonistic climate and enforced motivation of tax compliance. Additionally, the antagonistic climate is instable or slippery as it depends on permanent exertion of coercive power.

To change an antagonistic climate into a service climate, the measures of coercive power, such as controls and punishments, have to be combined with accepted, legitimate power. Once legitimate power is established, reason-based trust is likely to increase and, as a result, a service climate is established with voluntary tax cooperation. Tax authorities can improve their legitimacy by improving their services such as establishing professional and comprehensible tax procedures or web and telephone services in order to be perceived as motivated, competent and benevolent (Alm & Torgler, 2011). Moreover, the service climate is not depending on permanent enforcement, thus, it is relatively stable compared to the antagonistic climate.

A service climate is theoretically based on legitimate power and reason-based trust. It can be assumed that a service climate changes into a confidence climate and that voluntary cooperation changes into committed cooperation over the course of time and due to stable positive experiences (Verplanken, 2006). Cooperation founded on reason-based trust which is initially based on careful consideration of one's own risks and other's intents becomes automatic with routine and repeated positive experiences (Castelfranchi & Falcone, 2010; Dekker, 2004; Misztal, 1996; Nooteboom, 2002). Repeatedly positive experiences lead to the implicit expectation that the other party respects agreed norms and practices. Accordingly, reason-based trust decreases in the longer run, while correspondingly, implicit trust increases over time (Castelfranchi & Falcone, 2010). To promote a confidence climate, tax authorities could, for instance, establish contracts of fair play and long-term relationships with committed taxpayers (Adler, 2001; Alm & Torgler, 2011; Ouchi, 1979). Establishing fair play with enterprises and the guarantee of mutual collaboration on the basis of mutual trust are existing examples (e.g., Schepers, 2010; see also http://www.nltaxinternational.com/index.php/taxadvice/10; retrieved April 10, 2012).

It can be argued that relying solely on trust as in a confidence climate is far too optimistic in a social dilemma context since there always will be citizens tempted to engage in egoistic profit maximizing activities and hence, trusting authorities are not perceived as being able to enforce compliance. On the other hand, a climate of confidence is easily destabilized by the emergence of suspicion caused by power mechanisms (Kramer, 1999; Nooteboom, 2002). As a consequence, the confidence climate might be as instable and slippery as the antagonistic climate. Whereas the emergence of egoistic free-riders not threatened by coercive power might bring the confidence climate to a collapse, perceived power measures, depending on their quality and severity, can easily change the confidence climate into a service climate or an antagonistic climate if they trigger rational consideration of authorities' intentions or are perceived as hostile prosecution.

The attempt to describe the prerequisites of voluntary and committed cooperation is a worthwhile approach solving social dilemmas in assisting the transformation from an antagonistic climate to a climate of suitable services and confidence. Thus, long lasting and reoccurring experiences with legitimated authorities might convince recalcitrant taxpayers whose compliance is enforced to become responsible and self-determined citizens committed to the moral obligation of voluntary contribution to the common good.

Although the proposed model allows for several theoretical predictions and practical implications, it has some boundaries. It could be argued that legitimate power and reason-based trust represent similar concepts that are highly related. We see them as the two sides of the same coin. However, legitimate power is the perception of influence whereas reason-based trust is the decision to be vulnerable based on an evaluation of the influencing entity and its environment; therefore they are similar, but not identical. On the other hand, these well-established and related definitions of power and trust also highlight how, in fact, deeply connected power and trust are.

It is expected that the different interaction climates in a real life setting will never appear as sharply delineated as in theory. Therefore, future research should investigate the prevalence and overlaps of the different interaction climates, and how these affect tax compliance. Additionally, future research should not only consider trust in the authorities, but also trust in fellow citizens and believes about their motivation to cooperate (Eek & Rothstein, 2005). For instance, taxpayers may hold the opinion that they cooperate voluntarily, whereas other taxpayers only cooperate because they are enforced, and still other taxpayers cooperate out of commitment. Empirical studies should investigate whether such believes about others influence the dynamic between perceived power and trust.

The present paper provides a theoretical frame for future research. The presented conceptualizations of the dynamics between power and trust as well as subsequent tax climates, motivations to comply, and tax compliance serve as a theoretical basis for empirical studies. As an example, in countries differing in their overall tax climate, surveys among taxpayers could be conducted relating power perceptions and trust in authorities with compliance intentions and perceived tax climates, and motivations to comply. Experiments manipulating perceptions of cooperative climate and different qualities of power and trust with scenarios could be run to test the proposed relationships between the different qualities of power, trust, tax climates, motivations to comply, and tax compliance (Hofmann et al. 2014).

To conclude, by integrating of a wide range of psychological theories on cognition (Evans, 2008; Kahneman, 2003), leadership (Avolio & Bass, 1991; French & Raven, 1959), social and organizational climate (Adler, 2001; Lewin et al. 1939), compliance motivation (Kelman, 2006), situational influences (Alm & Torgler, 2006; Antonakis et al. 2003) and personal differences (Braithwaite, 2003) the SSF is extended. Starting off with the dynamic between power and trust, the interaction between tax authorities and taxpayers is explained which results in specific cooperative climates and corresponding forms of individual motivations to cooperate. Therfore, we are confident that the assumptions about the dynamics between power and trust are not only useful to understand and regulate tax behavior but can be transferred into other contexts related to cooperation in social dilemmas, managed by an authority. The present paper demonstrates that understanding the dynamic between power and trust can fuel research on cooperation in social dilemmas and can be utilized by authorities to foster voluntary and committed cooperation to guarantee the provision of public goods.
